# Azoturia

**Published:** 1903-05

**Authors:** F. H. Ames

**Affiliations:** Canton, Illinois


### AZOTURIA.

By F. H. Ames, D.V.S.,
CANTON, ILLINOIS.

AZOTURIA is the subject I have chosen, though it is one that has been written and discussed more than any other disease I can think of, but I am in hopes to learn more about it, and will be glad if any one here gains any information either from my paper or from the discussion which will follow. The etiology of azoturia is very generally agreed upon, though it is the opinion of many that a horse which has stood in the barn a long time is as subject to the disease as one which has been at work steady and has a few days of rest and on full feed. In my opinion it is wholly due to the horse being on full feed and at work, in which condition it will break down a large amount of tissue, and when it is allowed to stand for a day or more it will continue the breaking-down process, but will not carry it off, as would have been the case had it been at work; consequently the system becomes overloaded with uræmic products, and the condition follows which you are all familiar with.

There is not any danger of being mistaken in the diagnosis, as the symptoms are so marked, and the history of the case will make it impossible to confound it with any other disease.

Prognosis is a point where many can differ and be honest in their opinion. I think the literature on the subject gives the mortality too great, even though you cut out the cases which are slightly affected.

Treatment has been very satisfactory with me, though the treatment is not original with me.

On being called to see a horse with azoturia I get history of case and find out whether it has had previous attacks; I notice whether the horse has any manifestations of a weak circulation or weak kidneys, the legs being swollen is a common symptom. If these symptoms are present I give careful treatment in the way of antiseptics for kidneys.

I have found nothing to use in this trouble that does so well as salol in drachm doses. It is claimed by chemists that salol given internally will be changed in the kidneys to formaldehyde, and if so it has the proper action, as it will harden the kidneys and prevent the breaking down of these organs and the fatal termination which will follow.

When called to see a case I first give a physic ball, then follow up with one-ounce doses of sodii bicarbonas, either dissolved in water or wrapped in tissue paper and given as a bolus; repeat this every fifteen minutes until the patient has dried off and become quiet; then, if horse is down and unable to rise, draw its urine, wash out bladder with boric acid sol. I leave the man sufficient amount of the bicarbonas sol. to last, and give stimulants if much depression exists, and stimulate the horse with weak circulation with spirits nitrous ether and nux vomica. This I leave with the owner to give. Usually the horse will rise the second or third day.

I could burden you with the history of many cases, but will not, as you have given me your attention, for which I thank you one and all.

---

Dr. L. E. Willyoung, formerly of Buffalo, N. Y., is now located at Fort Sam Houston, San Antonio, Texas, as Veterinarian to the 2d Battalion F. Artillery.

